# Physicians' propensity to collaborate and their attitude towards EBM: A cross-sectional study

**DOI:** 10.1186/1472-6963-11-172

**Published:** 2011-07-25

**Authors:** Daniele Mascia, Americo Cicchetti, Maria Pia Fantini, Gianfranco Damiani, Walter Ricciardi

**Affiliations:** 1 Department of Public Health, Catholic University of the Sacred Heart, Largo F. Vito, 1-00168 Rome, Italy; 2 Department of Management, Catholic University of the Sacred Heart, Largo F. Vito, 1-00168 Rome, Italy; 3 Department of Public Health, University of Bologna, Via San Giacomo 12-40126 Bologna, Italy

## Abstract

**Background:**

The healthcare management literature states that physicians often coordinate their activities within and between organizations through social networks. Previous studies have also documented the relationship between professional networks and physicians' attitudes toward evidence-based medicine (EBM). The present study sought associations between physicians' self-reported attitudes toward EBM and the formation of inter-physician collaborative network ties.

**Methods:**

Primary data were collected from 297 clinicians at six hospitals belonging to one of the largest local health units of the Italian National Health Service. Data collection used a survey questionnaire that inquired about professional networks and physicians' characteristics. Social network analysis was performed to describe inter-physician professional networks. Multiple regression quadratic assignment procedures were performed to assess the relationship between self-reported attitudes toward EBM and clinicians' propensity to collaborate.

**Results:**

Physicians who reported similar attitudes toward EBM were more likely to exchange information and advice through collaborative relationships (β = 0.0198; p < 0.05). Similarities in other characteristics, such as field of specialization (β = 0.1988; p < 0.01), individual affiliations with hospital sites (β = 0.0845; p < 0.01), and organizational clinical directorates (β = 0.0459; p < 0.01), were also significantly related to physicians' propensity to collaborate.

**Conclusions:**

Communities of practice within healthcare organizations are likely to contain separate clusters of physicians whose members are highly similar. Organizational interventions are needed to foster heterophily whenever multidisciplinary cooperation is required to provide effective health care.

## Background

Supporting integration and sustaining collaboration among providers and professionals is of crucial importance for guaranteeing a multidisciplinary approach to health care. New organizational models have been developed to promote innovation, efficiency and quality of services by virtue of effectively pooling the unique expertise of professionals and organizations. Examples of the ways in which specialties and competencies of single professionals are integrated in organizational settings are represented by the creation of clinical directorates [1], the adoption of team working [2], or the use of disease management models [3].

In spite of the large diffusion of such models, coordination and work in health care increasingly occur through informal networks of relationships rather than through channels tightly prescribed by formal reporting structures or detailed work processes [4]. Physicians often establish interpersonal collaborative ties with colleagues to access and exchange clinical knowledge to solve daily problems or decide effective treatments for patients [5-7].

The relevance of social networks within communities of professionals has been recently investigated on empirical grounds by the healthcare management literature [8-12].

A number of mechanisms could explain the propensity of individuals to collaborate within organizations or to become part of a professional community. One of the most important mechanisms concerns "homophily" [13-15], whereby it is argued that individuals are more likely to create ties with peers who share the same traits because 'similarity of personal characteristics implies common interests and worldviews and best explains the formation of expressive ties based on interpersonal attraction' [16].

A growing stream of papers in the last few years has documented that physicians' attitude towards evidence-based medicine (hereafter EBM) is related to social factors such as professional networks [17-20]. In evidence since the 1990s, EBM incorporates individual clinical knowledge based on pathophysiology and prior experience with growing scientific evidence deriving from epidemiological and biostatistical ways of thinking as well as care for patient values [21,22].

Although the homophily mechanism can in principle operate with regard to any attribute that individuals may share [23], to our knowledge no surveys have investigated whether homophily in terms of physicians' attitude towards EBM relates to their propensity to establish collaborative network ties. Therefore, the aim of this study was to shed light on how the homophily of physicians in terms of their propensity to adopt EBM is likely to relate to collaborative network ties among them, taking into consideration other individual and professional features.

## Methods

### Research Setting and Data

The present observational study was conducted using a questionnaire survey of 329 physicians employed in six hospitals belonging to the local health authority (LHA) of Bologna, Italy, from February to November 2007. Responses to the questionnaire were requested within 3 months. Two quarterly recalls were sent to the physicians via email, and a final recall asked for a response within 1 month.

In Italy, LHAs aim to promote and protect the health of all resident citizens of a specific territory. The Italian National Health Service (INHS) is currently comprised of 145 LHAs. Based on considerations of efficiency and cost-effectiveness, each LHA may provide direct care through its own facilities or may commission the services of providers accredited by the system, such as independent public and private bodies.

The LHA of Bologna, which is one of the largest LHAs in Italy, serves approximately 800,000 individuals residing in 50 municipalities in the province of Bologna. The LHA employs around 8,400 people, including technical staff, nurses, and physicians, and has an annual budget of almost 1,300 million Euros. More than 80,000 hospitalizations occur annually.

In Bologna's LHA, hospital activities are carried out according to a matrix organizational model (Table 1). On the one hand, hospital activities are carried out in six hospital facilities: Bellaria, Bentivoglio, Budrio, Maggiore, Mazzacorati/Roncati, Porretta. On the other hand, these hospital services are provided by three clinical directorates (in Italian, "dipartimenti"). These are intermediate organizational establishments through which defined parts of larger hospitals' health services are managed [24]. The directorates were introduced as an institutional reference model for INHS healthcare organizations in the 1990s (laws 502/1992 and 229/1999), with the aim of reorienting activities toward healthcare processes by means of divisional units [25] in charge of strategic and organizational decision-making [1,26].

**Table 1:** Hospitals and clinical directorates physician staffing

	*Hospitals*							
***Clinical Directorates***	**Bellaria**	**Budrio**	**Maggiore**	**Porretta**	**Bentivoglio**	**Mazzacorati** **Roncati**	**Tot**	**%**
Neuroscience	78(78)	7(7)	64(64)	0	0	25(25)	174(174)	100
Oncology	64(41)	(0)	5(3)	0	6(4)	0	75(48)	64.0
Maternal Health	2(2)	2(2)	49(45)	6(6)	16(16)	5(4)	80(75)	93.7

Tot	144(121)	9(9)	118(112)	6(6)	22(20)	30(29)	*329(297)*	*90.3*
Compliance %	84.0	100	94.9	100	90.9	96.7		
								

Number of physicians on staff. In parentheses, number of survey respondents.

### Survey Instruments and Variables

Data were collected using a self-administered questionnaire containing three sections and 17 questions. The first section collected attributional data on clinicians: age, gender, hospital tenure, prior experience in the NHS, specialization, and managerial role. The second section was designed to collect data on information-exchange network relationships among clinicians. Like Burt [27], we used an egocentric social-network survey instrument to derive a list of people with whom the respondent had ties. Each physician was asked to name colleagues within and outside his/her hospital organization with whom he/she interacted through relationships based on the exchange of advice, and responses were combined in a summary network. We asked each respondent to characterize tie strength [28] with each nominated peer using a five-point scale. The third section of the questionnaire collected information about clinicians' attitude towards EBM. It included questions about respondents' perceptions of the availability of information and the possibility of accessing scientific evidence through corporate information-technology (IT) support.

Before distribution of the questionnaire to all physicians affiliated with the six hospitals and three directorates of the LHA, a pilot study was conducted with a convenience sample of physicians to ensure the practicability, validity, and correct interpretation of answers. In response to comments and suggestions made during the pilot study, confidentially was ensured by distributing questionnaires in individual envelopes and making them available online. Physicians completed questionnaires during breaks at work or at home.

Using survey relational data, we created an adjacency (or square) matrix containing information on the interpersonal collaborative ties between clinicians [29]. Each row/column listed physicians surveyed and intersecting cells represented the frequency (intensity) of interaction between pairs of individuals. We labeled this variable "Professional network (Valued)". A dichotomized version of this matrix, labeled "Professional network (dichotomized)", was additionally used in our analysis.

As in previous research [30], physicians' attitude towards EBM was investigated by asking how often in the past year they had used scientific evidence published in peer-reviewed biomedical journals to aid their practice of medicine. Responses were structured as "never," "rarely," "sometimes," and "often/very often."

We also included other demographic and work-profile variables such as gender, age, specialization, years since graduation from medical school, tenure in NHS and tenure in LHA-which overall represent individual seniority-and physicians' affiliation with hospital and clinical directorates. By including these variables we account for salient characteristics which likely affect physicians' behaviors and attitudes [11]. Such variables are also routinely reported in extant research exploring the relationship between EBM adoption and inter-physician collaboration [31,32]. We also included a dummy variable that characterized professionals' managerial responsibilities within the hospital system (managerial role). Following previous studies of the diffusion of innovations in the medical environment [33], we also considered physicians' scientific orientation by soliciting information about the number and authorship of papers they had published in the past 5 years. We observed that physicians within the surveyed organizations published co-authored papers with colleagues within and outside the LHA. Because co-authored publications reflect collaborations between individuals in the generation and exchange of new knowledge [34], the production of such publications likely influenced the propensity of physicians to establish collaborative ties in patient treatment. We thus controlled for the presence of co-authorship among physicians using the ISI Web of Knowledge database, which includes information on articles published in more than 20,000 scientific journals. We constructed a square matrix representing co-authorship linkages among surveyed clinicians. Each row/column listed physicians affiliated with the LHA and intersecting cells represented the presence or absence of co-authorship. The co-authorship matrix was constructed using ISI.exe software [35]. A final variable took into account the geographical distance (km) between physicians, computed using hospital-site affiliations. These distances were expressed in dyadic form in a square matrix.

### Statistical Analyses

We first performed social network analysis (SNA) to describe the inter-physician collaborative network. SNA is a method for the collection and analysis of data from multiple actors (or nodes: here, physicians) interacting through ties (or edges; here, collaboration in service provision) [30]. Because our data were relational (dyadic), physician pairs formed the primary unit of analysis.

Multiple regression quadratic assignment procedures (MR-QAP) were performed to identify predictors of inter-physician collaborative ties. MR-QAP is a combinatorial data-analysis procedure adopted routinely in social-network research [12,36,37]. The purpose of the MR-QAP is to regress a dependent relational matrix on one or more independent matrices, and to determine whether independent variables are significant predictors of the dependent variables. This procedure is used to model a social relation matrix using values of other relational matrices and control variables such as attributes of social actors. In the present analysis, the dependent variable was the interpersonal collaborative network, and the relational matrix representing geographical distances among physicians was treated as an explanatory variable. Because our data were relational, we transformed all covariates representing individual attributes. Specifically, continuous covariates (age, tenure etc.) entered the model as absolute differences between "sender" and "receiver" physician values. Smaller differences indicated greater similarity between physicians, and values of "0" indicated that the physicians were identical with respect to a given attribute. In other words, differences in continuous attributes measured the degree of homophily among members of dyads. A positive (negative) sign for continuous variables indicates that larger (smaller) differences, i.e. heterophily (homophily) of physicians, predicts positively (negatively) the propensity of physicians to establish collaborative ties with colleagues.

In contrast, individual attributes represented by categorical (affiliation to directorates, type of specialization etc.) and binary (gender, managerial role etc.) covariates were transformed and entered the model as binary variables, which take the value "1" if both members of the dyad belong to the "same" category, and the value "0" otherwise. For example, the variable Directorate (same affiliation) assumes values of "1" whether both physicians are affiliated to the same clinical directorate, whereas values of "0" signified that members of the dyad are affiliated to different directorates.

We performed MR-QAP analyses using the UCINET 6 software package [38]. The significance level accepted was p < 0.05.

## Results

**Figure 1:**
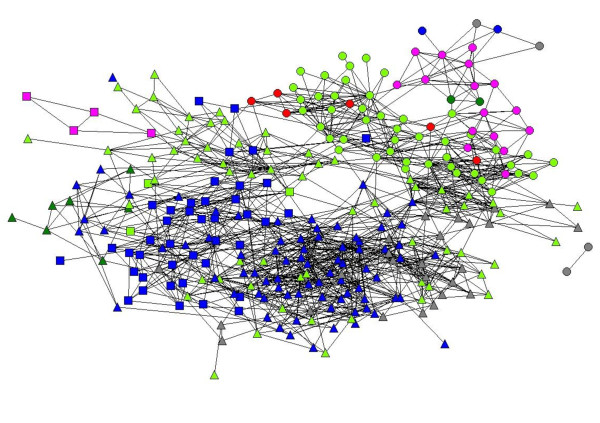
**Sociogram of Physicians' Professional Network**. (Directorate affiliation symbols: Neuroscience-Up Triangles, Oncology-Squares, Maternal Health-Circle. Hospital affiliation symbols: Bellaria-Blue, Porretta-Red, Bentivoglio-Pink, Budrio-Green, Maggiore-Light Green, Mazzocorati/Roncati-Grey).

An overall compliance rate of 90.3% (297 respondents) was achieved (Table 1). Because the dyad was the unit of analysis in the present study, the final sample consisted of 87,912 dyadic observations. Figure 1 depicts the network of collaborative relationships among 297 physicians in the six hospitals. Each circle (node) represents one physician in the dataset and each link (edge) represents an existing collaborative tie among node pairs. Node colors represent physicians' affiliation with LHA hospitals, and node shapes represent their affiliation with clinical directorates. Physicians' locations in Figure 1 were determined using a spring-embedding heuristic, multidimensional scaling algorithm, with proximity indicating the extent to which two physicians were connected directly and indirectly through mutual colleagues [39]. Table 2 describes the matrix representing the professional network and its dichotomized version. These descriptions relate primarily to the dependent network variable used in the present study. Table 3 summarizes the characteristics of the 297 physicians sampled.

**Table 2:** Descriptive Statistics of the Professional network

	Professional Network (Valued)	Professional Network (Dichotomized)·
Density (Mean) (%)	5.74	1.72
Std Dev	0.4685	0.1301
Sum	5,046	1,512
Variance	0.2195	0.0169
Minimum	0	0
Maximum	5	1
Reciprocated ties (%)	61.43	61.43
Symmetric Pairs (%)	97.34	97.77
No of dyads	87,912	87,912

• Dichotomized network where x'_ij _= 1 se x_ij _≥ 1, and x'_ij _= 0 otherwise.

**Table 3:** Characteristics of the 297 physicians

Attitude towards EBM, mean ± SD (range)	2.98 ± 0.56 (1-4)
Age, year, mean ± SD (range)	47.01 ± 8.01 (30-67)
Gender, No. M/F	158/139
Years since Graduation, year, mean ± SD (range)	27.05 ± 8.69 (7-55)
Field of Specialization, No. Physicians^· ^(%)	
Medical Specialties	210 (69.08)
Surgical Specialties	32 (10.53)
Obstetrics-gynaecology	39 (12.83)
Paediatrics	23 (7.57)
Tenure NHS, year, mean ± SD (range)	16.01 ± 9.74 (1-41)
Tenure LHA, year, mean ± SD (range)	10.95 ± 7.94 (1-37)
Managerial role, No. managers/professionals	51/246
Hospital affiliation, No. Physicians (%)	
Bellaria	121 (40.74)
Budrio	9 (3.03)
Maggiore	112 (37.71)
Porretta	6 (2.02)
Bentivoglio	20 (6.73)
Mazzacorati/Roncati	29 (9.76)
Directorate affiliation, No. Physicians (%)	
Neuroscience	174 (58.59)
Oncology	48 (16.16)
Maternal-Health	75 (25.25)
Number of Publications, mean ± SD (range)	4.27 ± 9.32 (0-83)
Co-authorship, mean ± SD (range)	0.01 ± 0.41 (0-1)
Geographical distance, km, mean ± SD (range	9.09 ± 12.68 (0-77)

Physicians may be specialized in more than one field.

Table 4 presents the results of the MR-QAP analyses, including estimates for both the dependent variables employed in the analysis.

**Table 4:** MRQAP estimating factors associated with the propensity of physicians to collaborate

Dependent Variable	Professional Network (Valued)			Professional Network (Dichotomized)·		
	**Estimate^†^**		**Significance**	**Estimate^†^**		**Significance**

Intercept	0.0000			0.0000		
Attitude towards EBM (same category)	0.0198	**	0.025	0.0201	**	0.020
Age (difference in)	0.0096		0.242	0.0089		0.261
Gender (same category)	0.0075		0.081	0.0080		0.077
Years since Graduation (difference in)	-0.0318	***	0.003	-0.0299	**	0.012
Field of Specialization (same category)	0.1988	***	0.000	0.1984	***	0.000
Tenure NHS (difference in)	0.0152		0.126	0.0156		0.146
Tenure LHA (difference in)	0.0030		0.377	0.0031		0.368
Managerial role (same category)	-0.0307	***	0.000	-0.0381	***	0.000
Hospital (same affiliation)	0.0845	***	0.000	0.0919	***	0.000
Directorate (same affiliation)	0.0459	***	0.000	0.0509	***	0.000
Number of Publications (difference in)	-0.0167	**	0.017	-0.0172	**	0.018
Co-authorship (same category)	0.1732	***	0.000	0.1782	***	0.000
Geographical distance	-0.0177		0.056	-0.0196	**	0.045

Observations (No of dyads)	87,912			87,912		
Multiple *R^2^*(Adj.)	0.314 (0.311)			0.312 (0.310)		
p-value	0.000			0.000		

Number of permutations: 5,000; ^† ^Standardized coefficients; *** p < 0.01; ** p < 0.05;

• Dichotomized network where x'_ij _= 1 if x_ij _≥ 1, and x'_ij _= 0 otherwise.

The positive coefficient for the parameter "Attitude towards EBM" (β = 0.0198; p < 0.05; Table 4) indicated that individuals belonging to the same category were more likely to collaborate. Overall, the propensity of physicians to create collaborative ties with their colleagues was related to similarities in the degree of self-reported EBM utilization. The dichotomized network variable produced the same result.

Among the control variables included in the analyses, continuous and categorical covariates were associated significantly with inter-physician cooperation. The "Years since Graduation", "Number of Publications", and "Geographical distance" covariates were negatively and significantly related to the propensity to collaborate. Specifically, physicians with a similar number of years since obtaining their degrees were less likely to cooperate (β = -0.0318; p < 0.01) and those who published similar numbers of papers in peer-reviewed journals were less likely to exchange information and advice (β = -0.0167; p < 0.05). Finally, physicians located greater distances from each other were less likely to cooperate on clinical matters (β = -0.0177; p < 0.1).

Five categorical covariates were associated significantly with the dependent variable (Table 4). The coefficients of the parameters for the "Field of Specialization", "Hospital", and "Directorate" variables were positive. Inter-physician collaboration was more likely to occur among individuals practicing the same medical specialty (β = 0.1988; p < 0.01). Differences in affiliation reduced the likelihood of inter-physician collaboration; physicians belonging to different hospitals (β = 0.0845; p < 0.01) and to different clinical directorates (β = 0.0459; p < 0.01) were significantly less likely to collaborate. The coefficient of the parameter for the "Managerial role" variable was negative, indicating that individuals with similar managerial roles were less likely to collaborate (β = -0.0307; p < 0.01). Finally, the "Co-authorship" variable was positively and significantly related to the professional network variable (β = 0.1732; p < 0.01), indicating that physicians who co-authored peer-reviewed papers were more likely to exchange advice and information during patient treatment.

Examination of the magnitude of standardized coefficients allowed us to assess the relative importance of predictors. Among the most influential homophily factors, the same field of specialization, co-authorship, affiliation with the same organizations, and similarities in physicians' attitudes toward EBM were most likely to result in inter-physician collaboration.

As shown in Table 4, results obtained using the dichotomized professional network as the dependent variable were qualitatively similar to those reported above.

## Discussion and Conclusions

The results of this study indicate that physicians with similar self-reported attitudes toward EBM were more likely to exchange information and advice through collaborative relationships. These results add new insights to previous research on communities of practice in healthcare [18,40-47].

The vast literature on methods for modeling the growth of networks beyond the domain of healthcare has provided a wealth of information on the ways in which communities are formed. Recent studies have applied social network analysis to explore the formation of communities within healthcare organizations [40-47], focusing the attention especially on the outcomes which derive from communities of professionals [17,18,8].

We focused on the identification of homophily mechanisms that predicted collaborative tie formation. Data from other contexts [13-15] led us to expect that the tendency of individuals to collaborate would be influenced by the relative degrees of homophily and heterophily. Homophily describes individuals' tendency to choose similar individuals as partners, whereas heterophily implies that actors are more prone to collaborate with different partners.

We found that homophily in physicians' attitude towards EBM was related significantly to collaborative behaviors undertaken within healthcare organizations. Although healthcare integration requires the combination of different competencies and specialties [40], we found that individuals with similar characteristics were more likely to interact. In particular, those with similar medical specialties and organizational affiliations were more likely to collaborate.

In accordance with previous studies [11], our results indicated that professional specialty was related strongly to inter-physician collaborative ties. This tendency may be due to the highly specialized education and training of physicians [48] and to several emerging organizational approaches in healthcare organizations. Clinical governance tools, which foster collaboration among peers favoring the introduction of peer-review evaluation [49], are typically based on homogeneity in medical specialties.

Our results also demonstrated a consistent relationship between organizational identities, defined in terms of affiliation with organizational units, and dyadic interactive activities underlying the complex network structures within organizational boundaries. In particular, physicians who were affiliated with the same hospital and located in the same geographic area were more likely to collaborate. The magnitude of the coefficients (β_hospital _= 0.0845; p < 0.01; β_directorate _= 0.0459; p < 0.01) indicated that proximity most strongly facilitated collaboration among organizational actors [9].

However, our findings indicated that homophily in some characteristics, such as "Years since Graduation", "Number of Publications", and "Managerial role", reduced physicians' propensity to collaborate. The negative impact of homophily for these three features on inter-physician collaboration may be interpreted as the presence of competition among physicians with equivalent roles within the organization [50].

Overall, our results have positive and negative implications for healthcare organizations. We documented the contribution of homophily to collaboration among physicians in clinical decision-making, which facilitates the transparency and reproducibility of clinical actions within organizations. However, we also found negative aspects representing the barriers that competitive role equivalence and specialties pose to integration within hospital organizations. It is also worth noting that an excessive degree of homogeneity may increase the risk of physicians clustering in homogeneous groups, thereby limiting the novelty of the knowledge they can share and foster.

For these reasons, the introduction of interventions that balance the documented tendency toward homophily with the need for integration among professionals with various scopes of knowledge and expertise is essential. Such interventions could be realized through multidisciplinary clinical audit meetings and the implementation of critical pathways that aim to cross the boundaries of physicians' specialties and professional experience.

The study has some limitations. The qualitative survey items may have been interpreted differently among respondents, influencing the consistency of their responses. In particular, we used self-reported EBM utilization as a measure of physicians' attitude towards EBM. Although this is not an objective approach to the study of physicians' orientations to EBM, many previous studies [30,51-53] have adopted this method and we are confident that our findings can be compared directly with those reported previously.

The study was also limited by the relatively low response rate we obtained for the social network analysis. Although the global response rate was high (90%), this rate fell to 64% for the oncology clinical directorate. However, this rate was well above the established minimum acceptance rates for such studies [29].

Another limitation was posed by our cross-sectional study design, which did not allow us to determine causality because all data were gathered at the same time. It provided, however, a hypothesis for causal links between EBM utilization and social collaborative relationships. Further longitudinal studies are necessary to clarify the relationship between homophily, defined in terms of EBM adoption, and social networks among professionals.

## Abbreviations

EBM: evidence-based medicine; INHS: Italian National Health Service; LHA: Local Health Authority; MR-QAP: Multiple Regression Quadratic Assignment Procedure; SNA: Social Network Analysis.

## Competing interests

The authors declare that they have no competing interests.

## Authors' contributions

DM and AC contributed to the conception of this paper; DM designed the study. GD and WR selected articles that met the inclusion criteria. DM and MPF extracted data and conducted the statistical analysis. All authors made substantial contributions to the interpretation of results and have seen and approved the final version.

## Pre-publication history

The pre-publication history for this paper can be accessed here:

http://www.biomedcentral.com/1472-6963/11/172/prepub
